# Perspectives on Aging Vestibular Function

**DOI:** 10.3389/fneur.2015.00269

**Published:** 2016-01-06

**Authors:** Eric Anson, John Jeka

**Affiliations:** ^1^Department of Otolaryngology Head and Neck Surgery, Johns Hopkins Medical Institutes, Baltimore, MD, USA; ^2^Department of Kinesiology, Temple University, Philadelphia, PA, USA; ^3^Department of Bioengineering, Temple University, Philadelphia, PA, USA

**Keywords:** aging, vestibular, VOR, balance, walking, functional testing

## Abstract

Much is known about age-related anatomical changes in the vestibular system. Knowledge regarding how vestibular anatomical changes impact behavior for older adults continues to grow, in line with advancements in diagnostic testing. However, despite advancements in clinical diagnostics, much remains unknown about the functional impact that an aging vestibular system has on daily life activities such as standing and walking. Modern diagnostic tests are very good at characterizing neural activity of the isolated vestibular system, but the tests themselves are artificial and do not reflect the multisensory aspects of natural human behavior. Also, the majority of clinical diagnostic tests are passively applied because active behavior can enhance performance. In this perspective paper, we review anatomical and behavioral changes associated with an aging vestibular system and highlight several areas where a more functionally relevant perspective can be taken. For postural control, a multisensory perturbation approach could be used to bring balance rehabilitation into the arena of precision medicine. For walking and complex gaze stability, this may result in less physiologically specific impairments, but the trade-off would be a greater understanding of how the aging vestibular system truly impacts the daily life of older adults.

## Aging and the Vestibular System

Many systems in the human body are adversely affected by the aging process, including the vestibular system. It has long been known that the number of vestibular hair cells is reduced in older adults compared to younger adults, independent of vestibular disease (1–4). The decline in hair cells is not uniform throughout the vestibular periphery. The saccule and utricle experience approximately a 25% reduction in hair cells, whereas semi-circular canals (SCCs) lose approximately 40% of their hair cells with age (5). Moreover, type I hair cells die off at a higher rate in the SCCs compared to the saccule and utricle, whereas type II hair cells experience similar rates of degeneration in the SCCs and otolith organs (3, 6–8). Utricular hair cells are more susceptible to age-related degeneration than saccular hair cells (3).

The size and number of neurons that make up the vestibular nucleus decrease by 3% each decade beginning around age 40 (9). The number of vestibular nerve fibers also declines with increasing age (10). Fewer vestibular sensory cells and neural pathways result in an age-related reduction in vestibular afferent signals to the central nervous system. There is also an associated reduction in the number of cerebellar Purkinje cells that contribute to modulation of vestibular afferents (11).

Paralleling the anatomical changes, most behavioral experiments have demonstrated a decline in functional vestibular tests [e.g., decreased vestibulo-ocular reflex (VOR) with increased age] (12–16). Fewer sensory cells in the SCCs result in a reduced capacity for detecting rotational head movements. In addition to reduced VOR gain, older adults also have shorter VOR time constants (12). Reduction in the vestibular time constant suggests the neural integrator as a potential site of age-related degeneration (13, 17). Dependence of the VOR on age appears to be variable as not all experiments demonstrate age-related decline in VOR gain (18, 19). VOR function measured by head impulse testing was impaired more often than otolith function based on vestibular-evoked myogenic potential (VEMP) testing for adults over 70 (20).

The functional consequence of fewer sensory cells in the otolith organs includes reduced sensitivity to gravity and linear acceleration (21, 22). Consistent with the decreased sensitivity of the saccule, older adults have smaller amplitude ocular and cervical VEMPs (23–27). Cervical VEMP response latencies are also longer and depend on a greater extent on stimulus volume to generate an effective response in older adults (27, 28). The optimal frequency for air conducted VEMPS also changes with increased age (25). Older adults display less ocular counter roll during slow roll tilt and also in response to galvanic vestibular stimulation consistent with reduced utricular responsiveness (29, 30). Linear VOR responses to fore-aft accelerations were smaller in older adults than in younger adults (31), demonstrating that the otolith responses to movement and to sound/vibration show a similar pattern of decline with age. The linear VOR has been implicated in anticipatory eye movements and a decline in otolith function may contribute to abnormal gaze stability during repetitive behaviors such as walking (32).

## Functional Impact of the Aging Vestibular System

It has been estimated that 30–35% of older adults suffers from vestibular dysfunction (33, 34). The most common type of vestibular disorder in the elderly is benign paroxysmal positional vertigo (BPPV) (35), likely due to fewer otoconia adhering to the saccule or utricle combined with alteration in calcium metabolism (22, 26, 36). Diagnosis of BPPV is based on stereotypical patterns of nystagmus and vertigo during positional testing, such as Hallpike–Dix testing (35, 37), supine head turns (38, 39), or deep head hanging (40). Routine medical screening for BPPV has been advocated for older adults due to the prevalence and ease of treatment (41).

Approximately one-third to one-half of the population over the age of 65 experiences an injurious fall annually (42, 43). Vestibular dysfunction results in balance impairments that frequently result in falls (44). Eighty percent of fallers in a recent study were found to have a vestibular impairment (45). Older adults experience more disequilibrium following nerve section associated with treatment of acoustic neuroma compared to younger adults (46). Persistent disequilibrium suggests that sensory reweighting may be more difficult with reduced vestibular input to the aging nervous system (47–49). Sensory reweighting involves prioritizing accurate and reliable sensory information over less reliable or less accurate sensory information for estimating body motion in space (50). Deviations in subjective visual vertical with age are consistent with reduced sensitivity of the otolith organs that lead to an increase in visual weighting to identify vertical (51, 52). Healthy older adults also demonstrate an increase in trunk sway velocity with age (53, 54). Older adults with abnormal utricular responses to whole body tilt have more variable medio-lateral sway relative to young adults, partly due to altered gravitational integration for postural control (29). Additionally, age-related changes in somatosensory function (reduced nerve conduction velocity), visual impairments, cognitive decline, and decreased strength may impact balance-related sensory integration for older adults who develop vestibular pathology (55–57).

Reduced capabilities in the aging vestibular system may impair the ability to rapidly detect changes in head acceleration and may contribute to slower walking as a self-protective strategy to prevent falls in older adults. Falls are known to occur most frequently during walking or transitions from sitting/standing to walking when head acceleration is higher (58). Abnormal SCC function [based on clinical head impulse test (HIT)] has been associated with slower gait speed and increased odds of falling in adults over 70 years old (59). By contrast, individuals with acute unilateral vestibular disease do not show a strong or consistent relationship between trunk velocity while walking and VOR gain (60). These inconsistencies may represent functional distinctions between VOR and vestibulo-spinal pathways despite neural convergence in the vestibular nuclei (61). Walking leg movement and trunk sway may receive different vestibular modulation as has been demonstrated for vision (62). Increased variability of double support time during walking has recently been reported for older female fallers with asymmetric responses to the post head shaking nystagmus test (63). Saccular function has also been shown to contribute to age-related changes in gait speed in healthy older adults (64). Slower gait speed may be a compensation related to postural abnormalities during a task when the base of support is dynamically changing (65), or to impaired visual stability at faster head speeds (66), or both. Differences in sample size, age, and pathology of vestibular dysfunction limit comparisons between these studies and highlight the need for additional studies to better understand the causal link between walking difficulties and age-related decline in SCC and otolith function.

The vestibular system has been linked to visuospatial function (67, 68), and individuals with vestibular loss experience difficulties with spatial navigation (69). Accurate spatial navigation depends on having a stable egocentric reference frame, and the vestibular system has been proposed as a source for that reference (70). Older fallers made significantly larger errors when performing a triangle walking task blindfolded, demonstrating a reduced ability to accurately perform spatial path integration (71, 72). Older adults have greater difficulty integrating multisensory cues for navigation than younger adults (73). Older adults are more likely to experience cognitive decline, and vestibular dysfunction mediates the decline in cognition associated with increased age (74). It is not clear to what extent age-related decline in spatial navigation measured when blindfolded relates to goal-oriented walking since path direction is influenced by vision, vestibular, and proprioceptive input (75).

However, many older adults with degenerating vestibular systems do not report imbalance or dizziness (13). Symptom reports do not consistently relate to either physiological (i.e., VOR) or perceptual assessments of vestibular function such as dynamic visual acuity (76–80). Further complicating the mismatch between symptoms and physiology, older adults may have anxiety/depression/fear of falling that exacerbates or mimics symptoms from age-related vestibular dysfunction (81–84). Due to limitations in vestibular diagnostic testing, clinicians may not be able to detect residual vestibular function in older adults with vestibular loss confirmed by current diagnostic testing (20, 85). Among older adults with severe vestibular loss canal function was impaired in 100% of individuals, but approximately 60% of those individuals demonstrate some degree of preserved otolith function (86). However, canal function evaluated by HIT does not require complete absence of function in order to be identified as pathologic (87, 88); therefore, there may also be preserved canal function in adults with age-related decline in vestibular function based on HITs. In addition to partially preserved vestibular function, inconsistent subjective reports may also be due to anticipatory mechanisms (89, 90), changes in lifestyle behaviors (91), or changes in multisensory reweighting (92, 93).

## Future Directions for Functional Vestibular Testing

Current vestibular assessments are very good at characterizing the reactivity of the peripheral vestibular sensory epithelium; however, they rely on artificial and unnatural stimuli to determine whether the vestibular system is working (79, 94, 95). The relevance of these artificial assessments to natural multisensory functional behavior is not always clear (96, 97). Even when there are associations between vestibular tests and functional activities, such as standing and walking, the direct causal link between walking and tests performed while sitting or lying down remains to be elucidated. Some tests such as calorics and clinical head impulse testing are very good at identifying abnormal vestibular function (88), but they may not be sensitive enough to identify slow decline in vestibular function associated with age (41). The range for clinically normal rotational VOR gain is 0.7–1.0, making it is unclear whether the measure of rotational VOR gain is adequate to capture age-related decline (14, 15, 98). Recently, more attention has been placed on corrective saccades resulting from head impulse testing as an additional method for identifying age-related change in vestibular-mediated gaze stability (99–101). Quantification of gaze stability based on compensatory saccades may prove to be more sensitive for identifying subtle age-related decline in vestibular function associated with aging. Since adaptive compensatory saccades contribute to gaze stability to a greater extent in response to vestibular pathology (102), new methods to quantify “global gaze stability” during natural behavior are needed that allow for multiple loci for neural control.

Current clinical balance assessments cannot specifically identify change in vestibular function as the primary contribution to balance problems for older adults. Perturbation-based evaluation of balance and sensory weighting allows for balance testing to move beyond descriptions of sway to mechanistic identification of abnormal multisensory integration (50, 103). This type of balance assessment has the potential to move beyond the standard approach to clinical balance assessment for older adults and bring balance rehabilitation closer to precision medicine. Major limitations to implement this level of precision diagnostics for balance include the expense of equipment, space for equipment, technical skills to perform the experiment, and interpret the experimental results. Additionally, the time needed to conduct these experiments may be clinically prohibitive. Future work in this field should focus on adapting perturbation style laboratory techniques for identifying mechanistic contributions to balance impairments into clinical settings (104), as well as controlled trials designed to target impaired balance mechanisms with rehabilitation strategies using a precision medicine approach. Clinical adaptations could include the use of body worn inertial sensors and head-mounted displays for visual stimulation to reduce equipment cost and space (105, 106). Electrical vestibular stimulation and tendon vibration could provide specific stimulation (103), rather than relying on non-specific effects encountered with foam surfaces. Demonstrating that equivalent results can be obtained in a shorter, more clinically friendly time frame is necessary before widespread clinical acceptance of this technique. Furthermore, task-specific balance assessment should not be restricted to standing balance. Body worn inertial sensors and smartphone technology can and should be leveraged to identify functional balance impairments during tasks, such as walking, obstacle crossing, and stair negotiation (60, 107, 108).

The ability to see clearly during head/body movement is important for many daily tasks, such as shopping, obstacle avoidance and manipulation, determining location/navigation by reading signs, and driving. The primary purpose of the VOR is to stabilize gaze during locomotion (109, 110). Oscillopsia, the apparent “jumping of objects … due to bobbing up and down of the head” degrades visual acuity during head motion making faces or reading signs/labels difficult or impossible to recognize (111–113). Oscillopsia can lead to reduced quality of life through reduction in activity participation, elevated economic burden, and self-imposed limitations on driving (86, 114). In contrast to seated tests of gaze stability, walking gaze stability depends on multiple sensory systems (vision, vestibular, and proprioception) for coordination of ocular muscles with postural muscles that control movement of the head (115–118). Characterizing overall gaze stability during walking would provide greater insight into actual functional problems experienced by older adults with reduced vestibular function. Overall gaze stability, despite being less physiologically specific, during a more natural behavior such as walking would be more informative about the daily life impact that vestibular decline has on older adults. In order to be more patient focused, future studies should move beyond the laboratory to evaluate functional gaze stability in natural settings during typical daily tasks. This is particularly relevant for studies attempting to link head impulse assessment of VOR gain to gaze stability during walking as peak head velocity during walking often exceeds the peak velocity of a head impulse (119). Portable lightweight gaze analysis systems could be sent home to capture a “day in the life” of an older adult or individuals with vestibular dysfunction.

Despite associations between VEMP tests and functional behaviors such as gait speed, the directionality and causality of those links remain unclear. Functionally relevant methods to evaluate otolith contributions to vestibulo-spinal control during standing and walking are needed (120, 121). Including assessments when the postural control system is under real or apparent threat, for example at heights, will also be important as vestibulo-spinal gain and postural sway are different under those conditions (121–123). Treadmills paired with virtual reality or head-mounted displays should be leveraged to evaluate spatial navigation for older adults. Immersive technology will facilitate simultaneous measurement of balance and walking ability, gaze stability, and eye movement control, while also tasking aspects of cognition, fear/anxiety, and ability to navigate through space. As new methods are devised to probe functional vestibular behavior, they will need to incorporate physiologically relevant vestibular stimulation for the SCCs and otoliths while also capturing the multiple systems influenced by the vestibular system (119, 124). A comprehensive and integrative approach to the evaluation of vestibular function should concurrently address gaze stability and postural control during functionally relevant standing and walking tasks.

## Author Contributions

EA and JJ conceived of the work. EA drafted the work. EA and JJ critically revised the work. EA and JJ approved the final version.

## Conflict of Interest Statement

The authors declare that the research was conducted in the absence of any commercial or financial relationships that could be construed as a potential conflict of interest.
